# Brazilian Society of Rheumatology and Brazilian Society of Clinical Pathology/Laboratory Medicine recommendation for serum uric acid test reports on patients undergoing treatment for gout

**DOI:** 10.1186/s42358-024-00415-6

**Published:** 2024-09-26

**Authors:** Geraldo da Rocha Castelar Pinheiro, Marco Antônio Araújo da Rocha Loures, Luís Eduardo Coelho Andrade, Fabiano de Almeida Brito, Leonardo de Souza Vasconcellos

**Affiliations:** 1 Serviço de Reumatologia, Universidade do Estado do Rio de Janeiro, Rio de Janeiro, RJ Brazil; 2 Sociedade Brasileira de Reumatologia, São Paulo, Brazil; 3 Departamento de Medicina, Universidade Estadual de Maringá, Maringá, Paraná Brazil; 4 Departamento de Medicina, Universidade Federal de São Paulo, São Paulo, SP Brazil; 5 Faculdade de Medicina, Universidade Federal de Minas Gerais, Belo Horizonte, MG Brazil; 6 Sociedade Brasileira de Patologia Clínica/Medicina Laboratorial, Rio de Janeiro, RJ Brazil; 7 Rheumatology Discipline, Pedro Ernesto University Hospital, Blvd. 28 de Setembro 77 Vila Isabel, Rio de Janeiro, RJ 20551-0301 Brazil

Gout is the most common form of inflammatory joint disease in humans [1, 2]. In recent decades, its prevalence has increased due to various factors: dietary habits, increased longevity, the use of hyperuricemic drugs, chronic kidney disease, and metabolic syndrome [1, 2]. Gout is a chronic disease caused by the deposition of monosodium urate (MSU) crystals in various tissues, manifesting as painful and potentially destructive arthritis in the context of hyperuricaemia [2–4]. Population studies in healthy individuals indicate a normal distribution of serum uric acid levels ranging from 3.4 to 7 mg/dL. However, hyperuricemia is defined as a serum urate concentration that exceeds its solubility at normal pH, body temperature, and sodium concentration [5]. The solubility threshold for uric acid is typically 6.8 mg/dL, but in peripheral joints, where body temperature is lower, the threshold is even lower. Therefore, during the treatment of gout, it is recommended that serum urate levels be maintained below 6 mg/dL [2–5]. Adequate control of hyperuricemia is crucial for preventing painful and disabling acute gout attacks. Both the prevention of precipitation and the resorption of already deposited MSU crystals depend on maintaining a serum uric acid level below its solubility threshold. In light of this, the Brazilian Society of Rheumatology and the Brazilian Society of Clinical Pathology/Laboratory Medicine recommend that serum uric acid test reports include a note stating that “*In patients undergoing treatment for gout*,* serum uric acid levels below 6 mg/dL are recommended”*. This therapeutic target (serum uric acid level < 6 mg/dL), which is well-supported in clinical practice, provides both physicians and patients with a clear and actionable goal, improving the monitoring and management of gout.

## Data Availability

No datasets were generated or analysed during the current study.
